# Dust exposure and the impact on hospital readmission of farming and wood industry workers for asthma and chronic obstructive pulmonary disease (COPD)

**DOI:** 10.5271/sjweh.3926

**Published:** 2021-03-01

**Authors:** Anne Vested, Henrik A Kolstad, Ioannis Basinas, Alex Burdorf, Grethe Elholm, Dick Heederik, Gitte H Jacobsen, Hans Kromhout, Øyvind Omland, Inger Schaumburg, Torben Sigsgaard, Jesper M Vestergaard, Inge M Wouters, Vivi Schlünssen

**Affiliations:** Department of Occupational Medicine, Danish Ramazzini Centre, Aarhus University Hospital, Aarhus, Denmark; Department of Public Health, Section for Environment, Occupation and Health, Danish Ramazzini Centre, Aarhus University, Aarhus, Denmark; Centre for Human Exposure Science, Institute of Occupational Medicine, Edinburgh, United Kingdom; Department of Public Health, Rotterdam, Erasmus Medical Center Rotterdam, The Netherlands; Division of Environmental Epidemiology, Institute for Risk Assessment Sciences, Utrecht University, The Netherlands; Department of Occupational Medicine, Danish Ramazzini Centre, University Research Clinic, Regional Hospital West, Jutland, Denmark; Department of Occupational Medicine, Danish Ramazzini Centre, Aalborg University Hospital, Aalborg, Denmark; National Research Centre for the Working Environment, Copenhagen, Denmark

**Keywords:** air pollutant, farmer, industry exposure matrix, lung disease, woodworker

## Abstract

**Objectives:**

It is still not well established how occupational air pollutants affect the prognosis of asthma or chronic obstructive pulmonary disease (COPD). This study uses nationwide Danish registers and quantitative dust industry exposure matrices (IEM) for the farming and wood industries to estimate whether previous year dust exposure level impacts hospital readmissions for workers diagnosed with asthma or COPD.

**Methods:**

We identified all individuals with a first diagnosis of either asthma (769 individuals) or COPD (342 individuals) between 1997 and 2007 and followed them until the next hospital admission for asthma or COPD, emigration, death or 31 December 2007. We included only individuals who worked in either the wood or farming industries at least one year during follow-up. We used logistic regression analysis to investigate associations between dust exposure level in the previous year and hospital readmission, adjusting for sex, age, time since first diagnosis, socioeconomic status, and labor force participation.

**Results:**

Asthma readmissions for individuals with low and high dust exposure were increased [adjusted rate ratio (RR_adj_) 2.52, 95% confidence interval (CI) 1.45–4.40] and RR_adj_ 2.64 (95% CI 1.52–4.60), respectively. For COPD readmission, the risk estimates were RR_adj_ 1.36 (95% CI 0.57–3.23) for low and RR_adj_ 1.20 (95% CI 0.49–2.95) for high exposure level in the previous year. For asthma readmission, stratified analyses by type of dust exposure during follow-up showed increased risks for both wood dust [RR_adj_ 2.67 (95% CI 1.35–5.26) high exposure level] and farming dust [RR_adj_ 3.59 (95% CI 1.11–11.59) high exposure level]. No clear associations were seen for COPD readmissions.

**Conclusions:**

This study indicates that exposure to wood or farm dust in the previous year increases the risk of hospital readmission for individuals with asthma but not for those with COPD.

Asthma and chronic obstructive lung disease (COPD) are prevalent obstructive lung diseases contributing substantially to morbidity and mortality worldwide (1). Organic dust exposure, most importantly from the farming and wood industries, is a suggested risk factor for both asthma and COPD (2, 3). However, how organic dust exposure affects individuals with asthma or COPD is not well established. We hypothesize that recent exposure to wood or farming dust is a risk factor for rehospitalization among individuals with asthma or COPD. We investigated this hypothesis in a longitudinal study using nationwide Danish administrative health and industry registers and quantitative dust industry exposure matrices (IEM) where hospital readmissions for asthma and COPD cases among ever farming and wood workers during follow-up were identified.

We aimed to investigate the association between previous year exposure to wood or farm dust and readmission for asthma or COPD.

## Methods

We recently published a study on the association between farming and wood dust exposure and COPD incidence using a population of Danish workers who worked in the farming or wood industries 1964–2007 and were born 1933–1977 (4). Using this population, we identified all individuals with a first hospital diagnosis of either asthma or COPD between 1997–2007 in the Danish National Patient Register (4) who were blue-collar workers in either the wood or farming industries after their initial asthma or COPD diagnosis. Employment data came from the Supplementary Pension Fund register, which provides annual information on employment (industry code) according to the Danish Industrial Classification of Economic Activities. Overall, 769 workers diagnosed with asthma (3777 person-years) and 342 with COPD (1369 person-years) were included.

The population was followed from the year after their first asthma or COPD hospital diagnosis (including emergency room and department visits) until the year of first hospital readmission for asthma or COPD, respectively, with censoring for death, emigration, loss to follow-up, 65 years of age, retirement or end of follow-up on 31 December 2007.

COPD was defined according to the International Classification of Diseases, 10^th^ revision (ICD-10) as emphysema (J43, J43.0, J43.1, J43.2, J43.8, J43.9) or other chronic obstructive pulmonary disease (J44, J44.0, J44.1 J44.8, J44.9). Asthma was defined by ICD-10 codes for asthma (J45, J45.0, J45.1, J45.8, J45.9) or status asthmaticus (J46, J46.9).

We established wood and farming industry-specific time-dependent IEM for inhalable dust exposure levels as described earlier (4). Briefly, arithmetic mean exposure levels were estimated from the WOODEX exposure database (5) and personal measurements from Danish farmers (6) both based on the inhalable dust fraction. The wood industry IEM included quantitative dust estimates for six industries: (i) sawmilling/planing of wood, (ii) manufacture of veneer boards/wood-based boards, (iii) manufacture of builders carpentry/joinery, (iv) manufacture of wooden packaging, (v) furniture industry, and (vi) carpenter and joiner business/construction. The farming IEM included estimates for crop-, cattle-, pig-, poultry-, mixed-, and fur-animal farming.

Each person-year of employment within the wood or farming industries during follow-up was assigned an exposure level based on the IEM, and follow-up years were further divided into three exposure groups [no (reference), low (>0–0.7 mg/m^3^) and high (>0.7 mg/m^3^)] according to the median exposure level. The majority of the reference group worked in other industries and a minority was unemployed (table 1).

**Table 1:** Baseline characteristics of 769 workers with an asthma diagnosis and 342 workers with a COPD diagnosis working in the Danish farming or wood industry during follow-up (1997–2007) by dust exposure intensity previous year.

Worker characteristics	Asthma population Dust exposure intensity previous year (mg/m^3^)				COPD population Dust exposure intensity previous year (mg/m^3^)			
	
	0	>0–0.70	>0.70–5.70	Missing	0	>0–0.70	>0.70–5.70	Missing
							
	17% (N=131)	39% (N=299)	40% (N=306)	4% (N=33)	13% (N=46)	41% (N=139)	42% (N=143)	4% (N=14)
	
	%	%	%	%	%	%	%
Age group (years)								
20–39	62	56	54		33	15	15	
40–49	24	24	23		26	30	19	
50–69	14	20	23		41	55	66	
Sex								
Male	85	76	84		89	76	89	
Female	15	24	16		11	24	11	
Labor force participation								
Yes	80	91	93		63	83	93	
No	20	9	7		37	17	7	
Socioeconomic status								
Top management	<5 ^a^	0	<2 ^a^		0	0	0	
Self-employed	7	3	<2 ^a^		<11 ^a^	<4 ^a^	<4 ^a^	
Higher and middle wage earners	<5 ^a^	4	<2 ^a^		<11 ^a^	<4 ^a^	<4 ^a^	
Employee at basic level	70	85	88		52	82	92	
Student/unemployed	20	8	7		37	13	5	
Missing	<5 ^a^	0	0		<11 ^a^	0	<4 ^a^	
Exposure source								
Wood	~65	76	56		~60	~80	~73	
Farming	~35	20	38		~40	~20	~27	
Wood and farming	-	4	6		-	-	-	

a Less than (<) is used to comply with the General Data Protection Regulation (GDPR) in order not to show <5 persons per cell. In such cases column percentages do not add up to 100%. Additionally, in order to comply with GDPR 10-year age groups have been combined into three groups in the table.

Associations between previous year dust exposure and hospital readmission for asthma or COPD were investigated with logistic regression analyses with person-years as the unit of analysis providing rate ratios (RR). We additionally performed stratified analyses for farming and wood workers separately excluding those with mixed exposure. All independent variables were lagged one year. Age (10-year categories), sex, time since first diagnosis (in years), socioeconomic status (SES), and labor force participation (yes/no) were included as covariates. Labor force participation was defined as a period, minimally 25% of the year, with no public benefit payment. We performed all analyses using STATA 13.0 (Stata Corp, College Station, TX, USA) on Statistics Denmark.

## Results

The study population spent 51% (asthma cases) and 57% (COPD cases), respectively, of their follow-up time in wood or farming related industries. The mean number of follow-up years was 3.9 (range 1–10) for asthma patients and 3.4 (range 1–10) for COPD patients. At baseline, the mean age was 39 [standard deviation (SD) 10] and 49 (SD 9) years for asthma and COPD patients, respectively. Most were men, had a basic level work, and a stable labor force participation. The majority of the follow-up years were in the high dust exposure groups. Workers who were not exposed at the start of follow-up were more likely to have a lower degree of labor force participation and basic level work (table 1).

In the main analysis, asthma readmissions were associated with previous year dust exposure, adjusted rate ratios (RR_adj_) 2.52 [95% confidence interval (CI) 1.45–4.40] for low and RR_adj_ 2.64 (95% CI 1.52–4.60) for high exposure level compared to no exposure in the previous year (table 2). RR_adj_ for COPD readmission were 1.36 (95% CI 0.57–3.23) for low and 1.20 (95% CI 0.49–2.95) for high exposure level in the previous year, compared to non-exposed in the previous year. For asthma readmissions, analyses stratified by type of dust exposure provided similar estimates as the main analysis for wood dust: RR_adj_ 2.38 (95% CI 1.23–4.60) and 2.67 (95% CI 1.35–5.26) for low and high exposure level previous year, respectively. For those exposed to farming dust only, the RR_adj_ were even higher: 4.03 (95% CI 1.20–13.52) and 3.59 (95% CI 1.11–11.59) (table 2). No clear association was seen for COPD readmissions, either for wood or farming dust. The small percentage (1.5% and 4%, respectively) of the asthma and COPD populations exposed to both wood and farming dust during follow-up were excluded in the stratified analysis.

**Table 2:** Rate ratios (RR) of asthma (N=769, 3777 person-years) and COPD (N=342, 1369 person-years) readmissions. Overall results and results stratified by farming or wood dust. In order to comply with the General Data Protection Regulation, cases and numbers of person-years for the stratified analyses of the COPD cases cannot be stated in the table.

Exposure level, (mg/m^3^)	Cases	Person-years	RR_crude_ (95% CI)	RR_adj_^a^ (95% CI)
**Overall**				
Asthma				
0	24	1863	1	1
>0–0.7	45	932	3.89 (2.35–6.42)	2.52 (1.45–4.40)
>0.7	49	982	4.02 (2.45–6.60)	2.64 (1.52–4.60)
COPD				
0	13	568	1	1
>0–0.7	17	375	2.03 (0.97–4.22)	1.36 (0.57–3.23)
>0.7	17	426	1.77 (0.85–3.69)	1.20 (0.49–2.95)
**Wood**				
Asthma				
0	18	1018	1	1
>0–0.7	34	697	2.85 (1.60–5.09)	2.38 (1.23–4.60)
>0.7	33	650	2.97 (1.66–5.32)	2.67 (1.35–5.26)
COPD				
0			1	1
>0–0.7			2.13 (0.92–4.94)	1.49 (0.52–4.28)
>0.7			1.42 (0.59–3.41)	0.90 (0.30–2.75)
**Farming**				
Asthma				
0	5	762	1	1
>0–0.7	9	164	8.79 (2.91–26.59)	4.03 (1.20–13.52)
>0.7	14	273	8.18 (2.92–22.94)	3.59 (1.11–11.59)
COPD				
0			1	1
>0–0.7			1.34 (0.24–7.45)	1.32 (0.19–9.08)
>0.7			3.03 (0.79–11.56)	2.61 (0.54–12.49)

a Adjusted for sex, age, years since first diagnosis, socioeconomic status, and labor force participation.

## Discussion

In this analysis, being exposed to wood and farming dust in the previous year more than doubled the risk of hospital readmission for asthma patients but not COPD patients. Analyses stratified by wood and farming dust exposure showed even higher risk estimates for farming-dust-exposed workers.

Studies suggest that hospital readmissions for asthma and COPD are related to the level of ambient air pollution (7). However, we are not aware of studies investigating how occupational dust exposure impacts hospital readmissions for workers with asthma or COPD. A 40% increased risk of COPD (but not asthma) exacerbations has previously been reported among COPD patients living within a radius of 500 m of a livestock farm (8). People with asthma more often report uncontrolled asthma in jobs with airborne exposures compared to jobs without (9), and ongoing occupational exposure has been associated with a poorer prognosis for individuals with asthma caused by occupational agents (10). Barely any knowledge on the impact of occupational exposures on COPD prognosis is available. However, a study from the US suggests a history of occupational exposures to be associated with a worse prognosis in patients with COPD (11).

In the current study, we focused on the impact of previous year exposure to wood or farming dust on hospital readmissions for asthma and COPD patients. For asthma, we realize that acute exacerbations, leading to hospital readmission, can be related to short-term peak exposure, however, we hypothesized that not only peak exposures but also dust exposure in the preceding months affects the prognosis, possibly due to low grade inflammation caused by ongoing exposure.

Even though the organic components in wood dust (mainly celluloses, hemicelluloses, lignin and low molecular weight organic and inorganic compounds) and farm dust (mainly organic material from plants, fodder, animals, microorganisms, and soil) are probably highly different, quite similar results were seen for the two types of dust.

A strength of this study is the use of comprehensive Danish employment and health registers combined with quantitative estimates of wood and farming dust exposure in a register-based cohort study with minimal risk of recall bias. A limitation is missing smoking information. However, for a subgroup of the cohort from which the wood and farming workers with asthma and COPD of the current study are identified, we observed no association between wood or farming dust and smoking (4). Therefore, we do not anticipate smoking to confound our findings. The observed null finding regarding COPD readmissions is likely explained by the healthy worker survivor effect, which was evident also in our recent publication on wood or farming dust exposure and risk of COPD (4). We assume severity of COPD has a large impact on work ability and that this may underestimate the effect of dust exposure, further supporting the healthy worker survivor effect.

Another limitation is that job task information cannot be accounted for in IEM. However, the farming and wood industry are well-defined entities where the probability of exposure is high and substantial variability in exposure within different farming and wood industries are well documented.

In conclusion, this study indicates that previous year exposure to wood or farming dust increases the risk of hospital readmission for individuals with asthma but not for individuals with COPD. In future studies more detailed information on severity of asthma and COPD are warranted.
